# Interleukin Expression after Injury and the Effects of Interleukin-1 Receptor Antagonist

**DOI:** 10.1371/journal.pone.0071631

**Published:** 2013-08-01

**Authors:** Connie S. Chamberlain, Ellen M. Leiferman, Kayt E. Frisch, Stacey L. Brickson, William L. Murphy, Geoffrey S. Baer, Ray Vanderby

**Affiliations:** 1 Department of Orthopedics and Rehabilitation, University of Wisconsin, Madison, Wisconsin, United States of America; 2 Department of Biomedical Engineering, University of Wisconsin, Madison, Wisconsin, United States of America; Rutgers University, United States of America

## Abstract

Ligament healing follows a series of complex coordinated events involving various cell types, cytokines, as well as other factors, producing a mechanically inferior tissue more scar-like than native tissue. Macrophages provide an ongoing source of cytokines to modulate inflammatory cell adhesion and migration as well as fibroblast proliferation. Studying interleukins inherent to ligament healing during peak macrophage activation and angiogenesis may elucidate inflammatory mediators involved in subsequent scar formation. Herein, we used a rat healing model assayed after surgical transection of their medial collateral ligaments (MCLs). On days 3 and 7 post-injury, ligaments were collected and used for microarray analysis. Of the 12 significantly modified interleukins, components of the interleukin-1 family were significantly up-regulated. We therefore examined the influence of interleukin-1 receptor antagonist (IL-1Ra) on MCL healing. Transected rat MCLs received PBS or IL-1Ra at the time of surgery. Inhibition of IL-1 activation decreased pro-inflammatory cytokines (IL-1α, IL-1β, IL-12, IL-2, and IFN-γ), myofibroblasts, and proliferating cells, as well as increased anti-inflammatory cytokines (IL-10), endothelial cells/blood vessel lumen, M2 macrophages, and granulation tissue size without compromising the mechanical properties. These results support the concept that IL-1Ra modulates MCL-localized granulation tissue components and cytokine production to create a transient environment that is less inflammatory. Overall, IL-1Ra may have therapeutic potential early in the healing cascade by stimulating the M2 macrophages and altering the granulation tissue components. However, the single dose of IL-1Ra used in this study was insufficient to maintain the more regenerative early response. Due to the transient influence on most of the healing components tested, IL-1Ra may have greater therapeutic potential with sustained delivery.

## Introduction

Ligament and tendon repair involves a complex series of coordinated events orchestrated by various cell types, cytokines and other factors. The repair process extends months to years and results in scar tissue mechanically inferior to native tissue. This is in clear contrast to tissue “regeneration” which would recapitulate the native tissue. Numerous approaches to stimulate a regenerative scenario have been attempted, including tissue-engineering, non-steroidal anti-inflammatories, ultrasonic, or electrical stimulation, but none have resulted in complete regeneration. In pursuit of a more regenerative outcome, it is important to understand the fundamentals of the normal healing process. Previous work from our lab identified a number of cellular, vascular, and molecular components integral to early healing [1]–[3]. Specifically, macrophages within the injured ligament peaked between day 3 and 5, while blood vessels primarily appeared between day 7 and 11 post-injury. The change in macrophage infiltration and angiogenesis during healing is modulated by the interleukin environment. Macrophages provide an ongoing source of cytokines, including IL-1α, IL-1β, IL-6, and TNF-α, and are responsible for modulating inflammatory cell adhesion and migration as well as fibroblast proliferation. [4] Ablation of macrophage-produced cytokines subsequently results in decreased fibroblast proliferation and ECM deposition. Consequently, ablation of macrophage-produced cytokines impedes healing and delays functional recovery. [5] Therefore, identifying the interleukins specific to ligament injury during peak macrophage activation and angiogenesis may elucidate the inflammatory mechanisms and subsequent scar formation.

In response to signals derived from damaged tissue and interleukins, monocytes/macrophages undergo reprogramming which leads to the emergence of macrophage subgroups with distinct phenotypes. Two broad subsets include the M1 (classically activated) and the resident M2 (further divided into the M2a, b, and c groups) macrophages. After injury, monocytes are recruited, enter the damaged tissue and differentiate into M1 mononuclear phagocytes. These newly recruited cells secrete pro-inflammatory mediators, such as IL-1, and participate in activation of various cytotoxic processes, including the respiratory burst, which creates extensive collateral damage.[6]–[8] An excessive M1 macrophage response may lead to aberrant inflammation and extensive collateral damage. In contrast to the M1 inflammatory macrophages, the M2 macrophages exhibit potent anti-inflammatory activity and may play important roles in wound healing and fibrosis. [9], [10] The M2 macrophages antagonize the M1 macrophage response, which may be pertinent for the activation of the wound healing and for restoration of tissue homeostasis. Recent studies have indicated that M1 macrophages can be converted into anti-inflammatory macrophages with a M2 wound-healing phenotype. [11], [12] M2 macrophages can then produce factors that induce myofibroblast apoptosis, serve as antigen presenting cells, and decrease the magnitude and duration of inflammation to promote wound healing.

The current study was undertaken to identify interleukins involved during peak macrophage up-regulation and angiogenesis during early ligament healing using microarray analysis. Based on preliminary microarray results, we hypothesized that blocking IL-1 activity via IL-1Ra administration would modulate the macrophage response during early healing to reduce fibrosis and scarring.

## Materials and Methods

### Normal MCL Healing Model

All procedures were approved by the University of Wisconsin Institutional Animal Care and Use Committee. All surgeries were performed using isofluorane, and all efforts were made to minimize suffering. Twenty-seven skeletally mature male Wistar rats (275–299 g) were used as an animal model to study normal MCL healing after surgical transection. A transected rather than torn MCL was used as an experimental model to create a uniform defect for healing. Rats (n = 18) were anesthetized via isofluorane and subjected to bilateral MCL transection. A 1 cm skin incision was made over the medial aspect the left and right stifles, exposing the sartorius muscle and underlying MCL. The mid-point of the MCL was completely transected and the muscular, subcutaneous and subdermal tissue layers were each closed with 4-0 Dexon suture. Animals were allowed unrestricted cage movement immediately after surgery. Another group of 9 animals did not undergo surgery and served as intact controls. At 3 and 7 days post-injury (n = 9/day), MCLs were snap-frozen and used for microarray analysis.

### Microarray Analysis

In order to identify interleukins regulated during peak macrophage and angiogenesis infiltration, MCLs (n = 9/day) were collected at day 3 and 7 post-injury and compared to interleukin expression of the intact MCL (n = 9) using microarray analysis. Tissue was processed as previously reported. [1] Briefly, snap-frozen MCLs were homogenized in Trizol and total RNA was isolated via column fractionalization using the RNeasy Total RNA kit (Qiagen, Valencia, CA). The quality and quantity of each RNA sample was analyzed on the Agilent 2100 BioAnalyzer using the RNA6000 NanoChip and the NanoDrop 1000 Spectrophotometer (Thermo Scientific, Wilmington, USA), respectively. Total RNA was transcribed to cDNA using an oligo dT primer containing the T7 RNA polymerase promoter. Microarray hybridization was performed by the University of Wisconsin Biotechnology Gene Expression Center (Madison, WI). Double stranded cDNA was purified and biotin-labeled in accordance with the Affymetrix gene analysis protocol (Affymetrix, Santa Clara, CA). cRNA was fragmented at 0.5 µg/µl final concentration and the size range of the cRNA before (0.1 kb and longer) and after (35–200 base fragments) was checked by agarose gel electrophoresis. The Affymetrix Rat 2.0 Genome GeneChips (Affymetrix, Santa Clara, CA) containing over 31,000 probe sets and representing 28,700 rat genes were used. A total of 9 chips were hybridized using 3 biological triplicates (containing 3 ligaments per sample) at 3 time points (intact, day 3 and day 7). GeneChips® (Affymetrix, Santa Clara, CA) were hybridized with biotin labeled ds-cDNA for 16 hours at 45°C. Following hybridization, the labeled samples were washed and exposed to streptavidin-phycoerythrin. Quantification of GeneChips was performed by Affymetrix GeneChip Operating Software (AGCC version 2.0). Microarray data were log transformed [log (base 2)] and the log ratio between the intact ligament with day 3 or day 7 was calculated and expressed as fold change. A gene was considered up or down regulated if the day 3 or day 7 samples were at least 2 fold different than the intact controls. Batch results were submitted to the Gene Expression Omnibus (GEO) repository (Accession number:GSE47676). For summary of microarray results not pertaining to the interleukins, refer to Chamberlain, et al., 2011. [1].

### IL-1Ra Experimental Model

In order to identify the influence of IL-Ra on MCL healing, 22 rats were randomly divided into 2 groups (n = 11 rats/treatment) and subjected to bilateral MCL transection (as described above). Treatment regime was based on previously published reports. [13] Thirty minutes prior to surgery, animals were administered 6.25 ng/ul IL-1Ra or PBS i.p. (IL-1Ra diluent; control) immediately before surgery and 3 hours post-surgery. At 5 and 11 days post-injury (n = 3 rats/trt/day) MCLs were measured for length, collected, and weighed; ipsilateral MCLs were used for histology/immunohistochemistry and contralateral MCLs for ELISA. Ligaments used for immunohistochemistry (IHC) were carefully dissected and immediately embedded longitudinally, in optimal cutting temperature (O.C.T.) medium for flash freezing. Tissue used for multiplex analysis was immediately snap-frozen. An additional set of MCLs were collected at day 11 (n = 5 rats/trt) and used for mechanical testing. Mechanical testing was not performed on day 5 because the ligaments were too structurally compromised for meaningful data with our testing method. Animals used for mechanical testing, were sacrificed and limbs were stored *in toto* at −70 C until used.

### IHC

In order to identify cellular and ECM changes within the healing MCL after IL-1Ra treatment, IHC and histology were performed on day 5 and 11 MCLs. Longitudinal cryosections were cut at a 5 µm thickness, mounted on Colorfrost Plus microscope slides and maintained at −70 C. IHC was performed on frozen sections using mouse monoclonal or rabbit polyclonal antibodies. Cryosections were fixed in acetone, exposed to 3% hydrogen peroxide to eliminate endogenous peroxidase activity, blocked with Background Buster (Innovex Biosciences, Richmond, CA) and incubated with rabbit or mouse primary antibodies. Sections were then incubated with biotin, and streptavidin-conjugated to horseradish peroxidase using the Stat Q staining kit (Innovex Biosciences, Richmond, CA). The bound antibody complex was visualized using diaminobenzidine (DAB). Stained sections were dehydrated, cleared, cover-slipped and viewed using light microscopy.

Mouse monoclonal antibodies to CD68 (1∶100, Abcam-Serotec, Raleigh, NC), CD163 (1∶100, Abcam-Serotec, Raleigh, NC), CD31 (1∶100, Abcam-Serotec, Raleigh, NC), α-smooth muscle actin (SMA; no dilution, Abcam-Serotec, Raleigh, NC), and Ki-67 (1∶100, Dako, Carpinteria, CA) were utilized to identify the classically activated macrophages (M1), alternatively activated macrophages (M2), endothelial cells, myofibroblasts and proliferating cells. To identify type I procollagen (straight; SP1.D8; Developmental Hybridoma, Iowa City, Iowa) and type III collagen (1∶8000, Sigma-Aldrich, St. Louis, MO) mouse antibodies were used.

After IHC staining, micrographs were collected using a camera assisted microscope (Nikon Eclipse microscope, model E6000 with an Olympus camera, model DP79). Within each stained tissue section, three images were captured within the granulation tissue (granulation tissue proximal edge, granulation tissue distal edge, center of granulation tissue), two images were captured away from the granulation tissue (proximal end and distal end of the MCL), and one image was captured within the epiligament. Two to three sections were counted per animal. Images captured for measurement of granulation tissue size, endothelial cells, myofibroblasts, type I procollagen, and type III collagen were quantified via Image J (NIH). Images of blood vessel lumen, proliferating cells, and M1 and M2 macrophages were quantified manually.

### Histology

Ligament cryosections were H&E stained to observe general morphology of the healing ligaments. After staining, images were captured and the granulation tissue regions were measured using Image J (National Institutes of Health, NIH). Granulation tissue area was normalized to the total MCL area and expressed as the percent normalized granulation tissue.

### Multiplex Assay

To identify the influence of IL-1Ra on MCL cytokine production, a rat 10-plex Luminex assay (Life Technologies, Grand Island, NY) was performed using day 5 and 11 MCLs. MCLs were washed in Cell Wash Buffer (Bio-Rad, Hercules, CA) placed in Navy Bead Lysis Kit tubes containing a 0.9–2.0 mm stainless steel bead blend, 3.2 mm stainless steel balls (Next Advance, Averill Park, NY) and Lysing Solution (Bio-Rad, Hercules, CA). Samples were homogenized via Bullet Blender (Next Advance, Averill Park, NY) for 10 min. Supernatant was collected and used for BCA (to determine total protein concentration) and subsequent Multiplex analysis. Multiplex cytokine assays (Life Technologies, Grand Island, NY) were performed according to the manufactures instructions. Diluted capture bead solution was vortexed, sonicated and added to each well. Standards, samples, spleen (serving as positive control), and lysis buffer (serving as negative control) were added to the wells and incubated in the dark, overnight at 4 C on a plate shaker. The next day, samples were washed, incubated with biotinylated detector antibody, and streptavidin-RPE solution. After washing and re-suspension in working wash solution, samples were read on a Luminex 200 instrument (Luminex, Austin TX). Ten proteins were included in the platform, including IL-1α, IL-1β, interleukin-10 (IL-10), interleukin-2 (IL-2), interleukin-12 (IL-12), interleukin-4 (IL-4), interleukin-6 (IL-6), tumor necrosis factor-α (TNF-α), interferon-γ (IFN-γ), and granulocyte macrophage-colony stimulating factor (GM-CSF). Assessment of the Lumenix assay was performed by verifying each standard curve point was within 80–120% recovery, and 2 standard deviations above background. Cytokine concentrations were normalized to protein concentration and expressed as ng/mg.

### Mechanical Testing

In order to test the functional mechanical properties of the healing MCL after IL-1Ra treatment, day 11 ligaments were mechanically tested. Pull-to-failure testing was performed as previously described by Provenzano et al. ^14–16^ After sacrifice the MCL was removed with both femoral and tibial insertion sites and the surrounding tissue was carefully excised with special care taken to avoid damaging the insertion sites. During preparation, the femur-MCL-tibia (FMT) complex was kept hydrated using PBS. The width and thickness of the ligament was measured optically and the cross-sectional area for the ligament was estimated assuming an elliptical cross section. The FMT complex was mounted in a custom made testing bath and mechanical testing machine. Optical markers were applied to the ligament on the insertion sites and the displacement was recorded optically. The ligament was pre-loaded to 0.1 N and subsequently preconditioned (cyclically loaded to 1% strain for 10 cycles). Dimension measurements for the ligament were recorded at the pre-load. The ligament was then pulled to failure at a rate of 10% strain per second. Failure force was recorded as the highest load prior to failure of the ligament and failure stress was calculated by dividing the failure force by the initial cross-sectional area of the ligament. The slope of the linear portion of the line relating stress to elongation was used to calculate a normalized stiffness parameter.

### Statistical Analysis

Microarray data were analyzed using the two-sample Welch t-test to determine significance per gene. To minimize the false discovery rate, the Storey q-value method was performed and significance was based on q <.01. Any Q-value less than or equal to 0.01 per gene was considered significant. Paired t-tests were used to evaluate differences between intact and day 3, intact and day 7, or day 3 and day 7. A critical value of.05 was considered as the criterion to select a significant fold change in gene expression between days. Data are presented as fold change of the means of day 3 and day 7 from the intact ligament values (fold change ± S.D.). For the IHC results MCL regions were subgrouped (granulation tissue, outside of granulation tissue, and total MCL) to identify any spatial differences of each factor measured. A one-way analysis of variance (ANOVA) was then used to examine treatment and/or day differences for the IHC data, as well as the multiplex and mechanical data. If the overall p-value for the F-test in ANOVA was significant, post-hoc comparisons were performed using the Fisher’s LSD method. Experimental data are presented as the means ± S.E.M. All p-values reported are two sided. P<0.05 was used as the criterion for statistical significance. Computations and figures were performed using KaleidaGraph, version 4.03 (Synergy Software, Inc., Reading, PA).

## Results

### Microarray Results to Identify Interleukins (Il)

**Figure 1:**
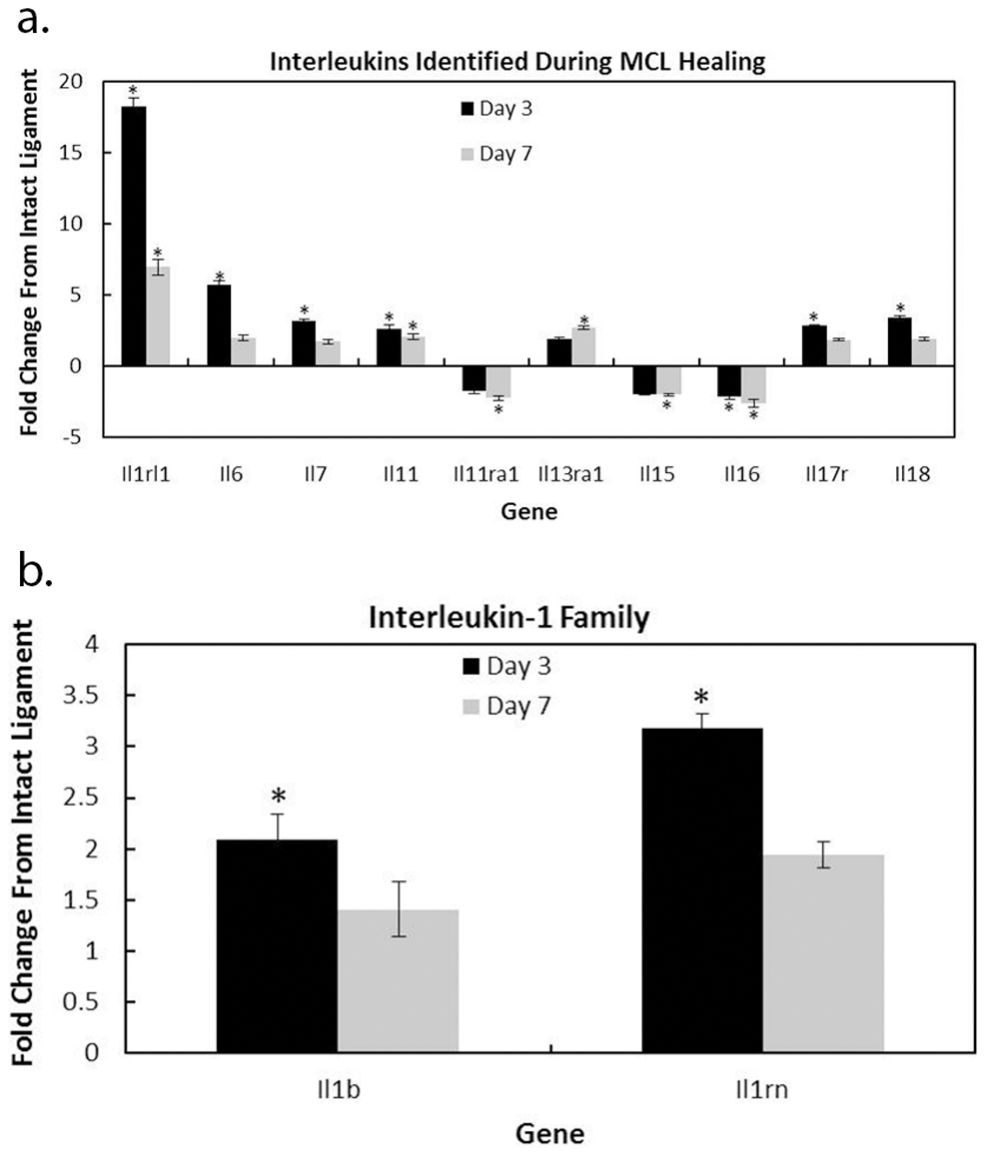
Microarray results identifying specific interleukins modulated at days 3 and/or 7 post-injury. A total of 12 interleukins were significantly modulated (2 fold greater than normal and q-value <.05) compared to the intact control. Of those 12, 6 were up-regulated at day 3 only (il6, il7, il17r, 1l18, il1b, and il1rn), 1 up-regulated at day 7 only (il13ra1), 1 down-regulated at day 7 only (il11ra1), 2 up-regulated at both day 3 and 7 (il1rl1, il11), and 1 down-regulated at both day 3 and 7 (il16). Interleukins regulated during healing were both pro- and anti-inflammatory (a). Members of the il1 family were significantly regulated with healing (b). Microarray data are compared to the normal intact ligament and expressed as fold change ± S.D. * indicate fold change ≥2 from the intact control.

Normal healing of the MCL 3 and 7 days post-injury resulted in significant modulation of 12 MCL-derived interleukins (Fig. 1; Table 1). Of those *Il*s, six resulted in a 2 fold or greater difference from the intact MCL at day 3, three at day 7, and three on both day 3 and day 7. Interleukin-1 receptor like-1 (*Il1rl1*) was the most prevalent interleukin up-regulated on both day 3 and 7. *Il11* and *Il16* were also significantly up- and down-regulated, respectively, on both days. Interleukins up-regulated at day 3 included *Il1b, Il1rn, Il6, Il7, Il17r,* and *Il18* (no interleukins were down regulated at day 3). Those ILs regulated at day 7 included *Il11ra1*, *Il13ra1,* and *Il15*. These results indicate that interleukins with a predominantly pro-inflammatory role play an active role during early normal ligament healing. Based on the time of MCL collection at 3 and 7 days, most of the interleukins likely originate from the macrophages.

**Table 1:** Microarray results identifying the interleukins significantly modulated during early healing.

Accession Number	Interleukin	Pro or anti-inflammatory	Q-value	d3 vs intact q-value	d7 vs intact q-value	d3 vs d7 q-value	d3 foldchange	d7 foldchange
1387273_at	Interleukin 1 receptor-like 1 (*I1rl1*)	Pro-	0.001	0.010	0.029	0.004	18.282	6.963
1369191_at	Interleukin 6 (*Il6*)	Anti-	0.000	0.006	0.036	0.000	5.742	1.989
1369208_at	Interleukin 7 (*Il7)*	Pro-	0.000	0.001	0.010	0.005	3.140	1.706
1369534_at	Interleukin 11 (*Il11*)	Anti-	0.003	0.009	0.027	0.239	2.619	2.058
1370331_at	Interleukin 11 receptor, alphachain 1 (*Il11ra1*)	Anti-	0.001	0.023	0.007	0.022	−1.753	−2.246
1370728_at	Interleukin 13 receptor, alpha 1 (*Il13ra1*)	Anti-	0.000	0.005	0.000	0.009	1.932	2.707
1368375_a_at	Interleukin 15 (*Il15*)	Pro-	0.000	0.000	0.001	0.792	−1.990	−2.030
1376895_at	Interleukin 16 (mapped; *Il16)*	Pro-	0.002	0.046	0.022	0.048	−2.124	−2.623
1373611_at	Interleukin 17receptor (predicted; *Il17r*)	Pro-	0.000	0.001	0.003	0.000	2.818	1.856
1369665_a_at	Interleukin 18 (*Il18*)	Pro-	0.000	0.001	0.009	0.003	3.369	1.898
1398256_at	Interleukin 1 beta (*Il1b*)	Pro-	0.007	0.038	0.169	0.019	2.086	1.407
1387835_at	Interleukin 1 receptorantagonist (*Il1rn)*	Anti-	0.000	0.001	0.007	0.001	3.186	1.946

Column 3, “Q-value” indicates the overall statistical significance. Columns with “d3 vs intact”, “d7 vs intact” or d3 vs d7” q-values (columns 4–6) are pairwise comparisons between different days of injury. The “d3-“ or “d7 fold change” columns (7–8) indicate fold differences when compared to the intact MCL.

### IL-1Ra Injury Model

**Figure 2:**
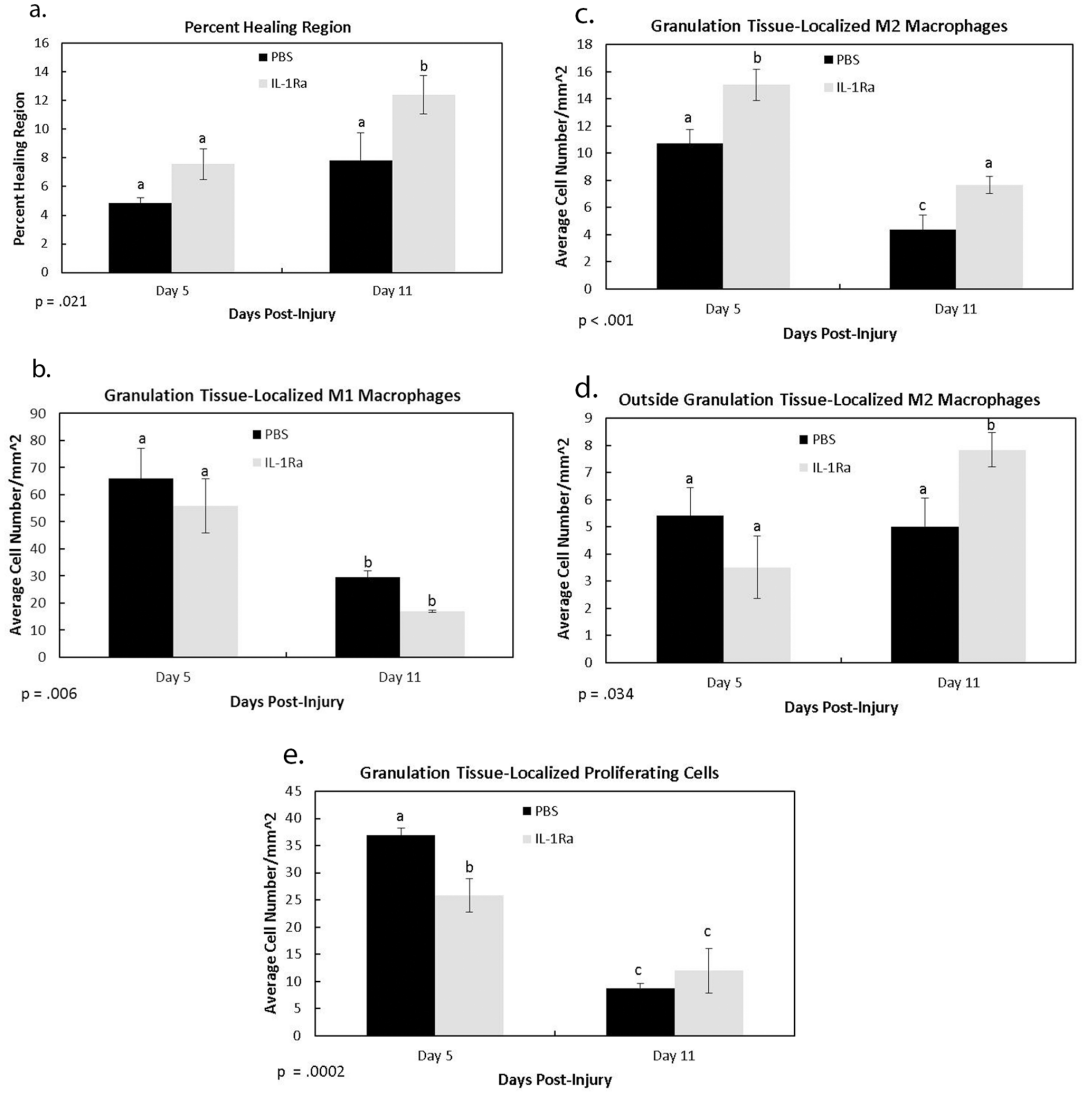
Immunohistochemistry results of selected cellular factors regulated by IL-1Ra. Graph of the percent healing region (a), granulation tissue localized M1 macrophages (b), granulation tissue-localized M2 macrophages (c), M2 macrophages outside the granulation tissue (d), and granulation tissue-localized proliferating cells (d), at 5 and 11 days post-injury after PBS or IL-1Ra treatment. On day 11, IL-1Ra significantly increased the percent healing region (a). No differences within the MCL were observed at any other points. Although no differences were observed with M1 macrophages (b), IL-1Ra treatment increased M2 macrophage numbers at day 5 and/or day 11 within (c) and outside of the granulation tissue (d). Proliferating cells were significantly reduced at day 5 (e). ^a,b,c^ indicates within a graph, bars without a common superscript letter differ (results of Fisher’s LSD post-hoc pairwise analysis, p<.05). Values are expressed as mean cell numbers ± S.E.M.

**Figure 3:**
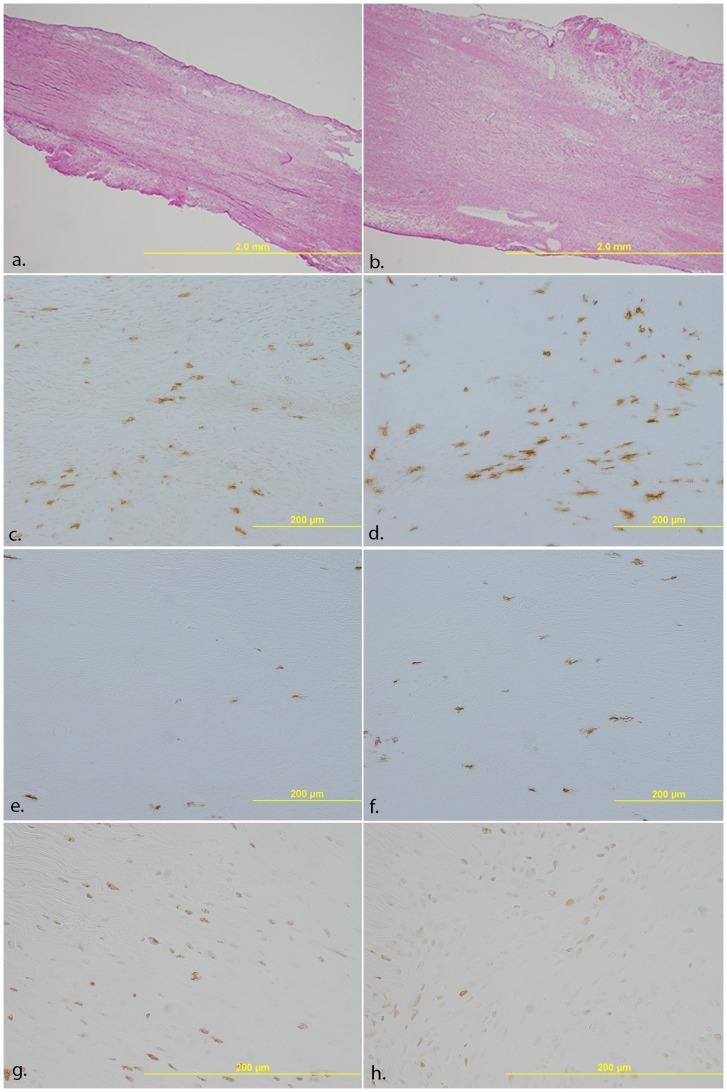
Representative micrographs of the healing MCL after PBS or IL-1Ra treatment. Micrographs demonstrate H&E staining (a–b), and immunohistochemistry of M2 macrophages (c–f), and proliferating cells (g–h) after PBS (left column) or IL-1Ra (right column) treatment. H&E staining of the day 11 MCL indicating larger granulation tissue size (a–b). M2 macrophages 5 days- (c–d) and 11 days post-injury (e–f) increase in number after IL-1Ra treatment. In contrast, proliferating cells decrease on day 5 (g–h) after IL-1Ra administration.

To further analyze the influence of interleukins on MCL healing IL-1Ra, a protein that binds IL-1 receptor to inhibit IL-1 activation, was administered to a rat MCL injury model. IL-1 was targeted based on its identification via microarray and its established pro-inflammatory influences in various pathologies. To first determine if IL-1Ra modulated wound healing size, granulation tissue area from H&E stained MCLs were sectioned and normalized to total MCL area (Figs. 2; 3a–b) indicates no change in granulation tissue size between PBS (4.86% ±.35) and IL-1Ra (7.57% ±1.07) at day 5. On day 11, IL-1Ra (12.40% ±1.33) significantly increased (p = .021) area of granulation tissue compared to the control (7.83% ±1.92).

### IHC of Granulation Tissue Factors

**Figure 4:**
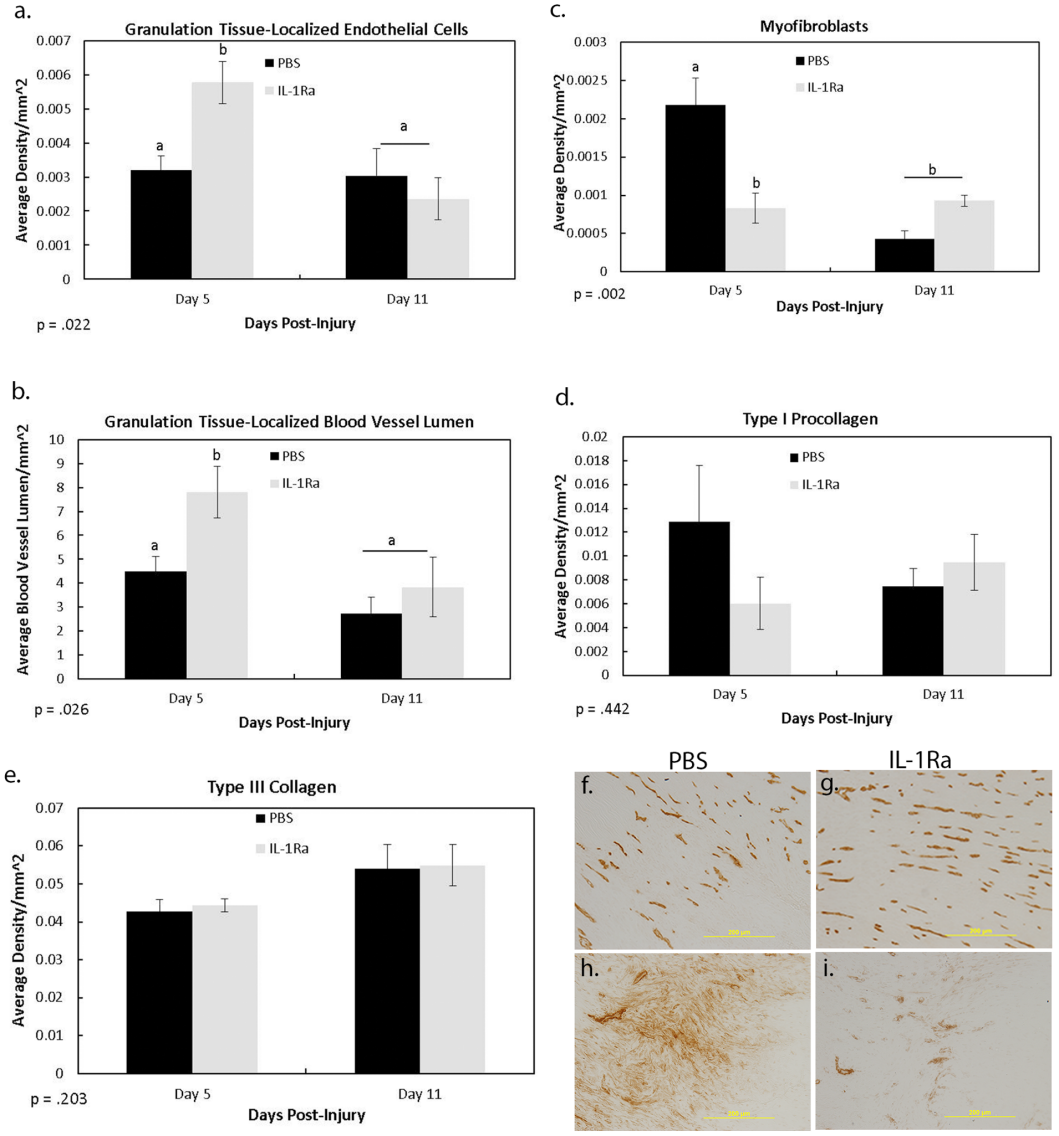
Immunohistochemistry results of cellular and ECM factors regulated by IL-1Ra. Immunohistochemistry results showing the effects of PBS and IL-1Ra on granulation tissue localized endothelial cells (a), granulation tissue-localized blood vessel lumen (b), total myofibroblasts (c), type I procollagen (d), and type III collagen (e) at 5 and 11 days post-injury. IL-1Ra significantly increased the number of endothelial cells and blood vessel lumen on day 5 (a–b), but not day 11. In contrast, IL-1Ra significantly decreased the number of myofibroblasts at day 5 (c). No differences within the MCL were observed on day 11 (c). Regardless of day, no treatment differences were observed with type I procollagen (d) and type III collagen (e). ). ^a,b^ indicates within a graph, bars without a common superscript letter differ (results of Fisher’s LSD post-hoc pairwise analysis, p<.05). Values are expressed as mean density ± S.E.M. Representative micrographs of endothelial cells/blood vessel lumen (f–g) and myofibroblasts (h–i) 5 days post-injury after PBS (left column) and IL-1Ra (right column) treatment.

Because IL-1 has been demonstrated to stimulate an inflammatory reaction after injury, components of inflammation and granulation tissue formation were monitored, including the M1 and M2 macrophages, proliferating cells, endothelial cells, blood vessel lumen, and myofibroblasts. Regardless of treatment, both M1 and M2 macrophages were significantly higher at day 5 than day 11 (Fig. 2). Treatment with IL-1Ra did not significantly change the M1 cell numbers (Fig. 2b). In contrast, treatment with IL-1Ra significantly increased the granulation tissue-localized M2 macrophages at both day 5 (10.72±1.01 vs 15.04±1.15 cells/mm^2^) and 11(4.39±1.06 vs 7.67±.63 cells/mm^2^) post-injury (Figs. 2c; Fig. 3c–f). M2 macrophages located outside of the granulation tissue were also significantly increased at day 11 after IL-1Ra treatment (5±1.06 vs 7.83±.63 cells/mm^2^; Fig. 2d). Immunohistochemistry of proliferating cells indicated a significant increase in cell number at day 5 vs day 11. However, treatment with IL-1Ra reduced the number of proliferating cells at day 5 (36.96±8.72 vs 25.89±3.07; Figs. 2e; 3g–h). No significant changes in proliferative cell number were observed outside the granulation tissue or epiligament. Endothelial cells and blood vessel lumen identified within the granulation tissue were increased in number after IL-1Ra treatment at day 5 whereas no effects were observed on day 11 (Figs. 4a–b; f–g). Similar to the proliferating cells, day 5 myofibroblasts were decreased with IL-1Ra treatment throughout the entire ligament (2×10^−3^±0.4×10^−3^ vs 0.8×10^−3^±.2×10^−3^; Fig. 4c, h–i). Results were not significant at day 11.

### Immunohistochemistry of Collagens

Since a number of granulation tissue localized factors were significantly modified by IL-1Ra, type I procollagen and type III collagen were measured to determine if any ECM factors were likewise changed during ligament healing. Fig. 4d–e indicates no significant change in either type I procollagen or type III collagen after IL-1Ra treatment.

### Multiplex Results

**Figure 5:**
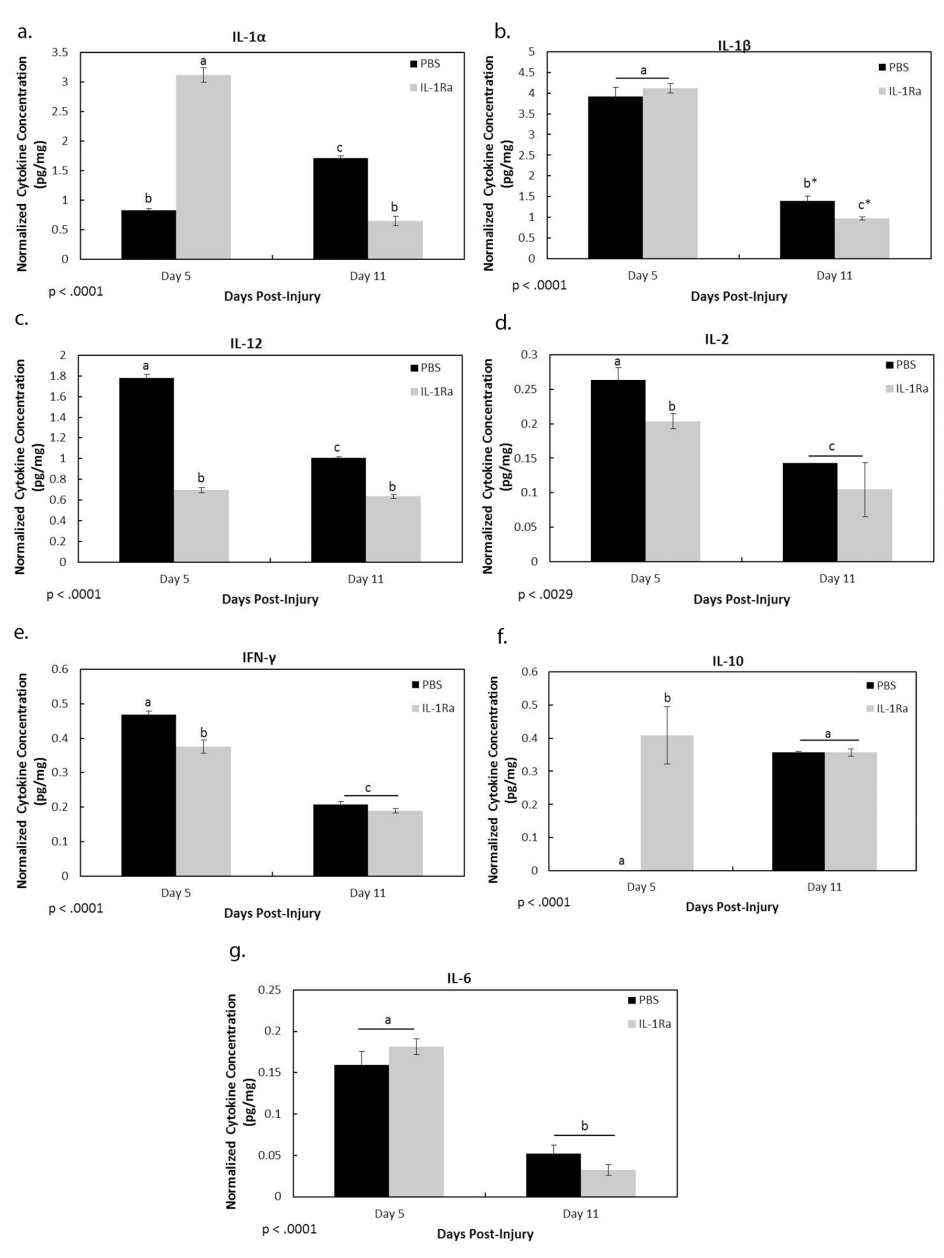
Luminex results of cytokines regulated by IL-1Ra. Graphs show the effects of PBS and IL-1Ra on MCL-derived IL-1α (a), IL-1β (b), IL-12 (c), IL-2 (d), IFN-γ (e) IL-10 (f), and IL-6 (g) production. Blockade of the IL-1 activation resulted in an accumulation of IL-1α (a) and IL-1β (b). By day 11, inhibition of the IL-1R resulted in a down-regulation of IL-1α (a) and tended to decrease IL-1β (b; p = .056). IL-1Ra significantly decreased the pro-inflammatory cytokines, IL-12 (c), IL-2 (d), and IFN-γ (e) on day 5. IL-12 levels remained significantly different after IL-1Ra on day 11. The anti-inflammatory cytokine, IL-10 was up-regulated at day 5 after IL-1 inhibition (f). IL-6 was not significantly influenced by treatment (f). Beneath graph, p value indicates ANOVA results. ^a,b,c^ indicates within a graph, bars without a common superscript letter differ (results of Fisher’s LSD post-hoc pairwise analysis, p<.05). *indicates p value.056. Values are expressed as normalized cytokine concentration (ng cytokine/mg tissue weight) ± S.E.M.

The previous IHC results identified the cells influenced by IL-1Ra treatment during MCL healing. The next experiments examined the influence of IL-1Ra on cytokine production by the cells of the MCL. As Figure 5 indicates, IL-1α (PBS, 0.83±.03 pg/mg vs. IL-1Ra, 3.11±.12 pg/mg; Fig. 5a), but not IL-1β (PBS, 3.92±.22 pg/mg vs. IL-1Ra, 4.12±.12 pg/mg; Fig. 5b), was up-regulated at 5 days post-injury after IL-1Ra treatment, suggesting that IL-1Ra may have blocked IL-1 receptor activation at day 5, but IL-1 production continued. By day 11, IL-1Ra treatment significantly reduced IL-1α (PBS, 1.72±.03 pg/mg vs. IL-1Ra, 0.65±.08 pg/mg) and tended (p = .056) to reduce IL-1β (PBS, 1.39±.12 pg/mg vs. IL-1Ra,.97±0.04 pg/mg). On day 5, the pro-inflammatory cytokines, IL-12 (Fig. 5c; PBS, 1.78±0.03 pg/mg vs. IL-1Ra, 0.70±0.02 pg/mg), IL-2 (Fig. 5d; PBS, 0.26±0.02 pg/mg vs. IL-1Ra.20±0.01 pg/mg), and IFN-γ (Fig. 5e; PBS,.47±.01 pg/mg vs. IL-1Ra,.38±.09 pg/mg) were significantly down-regulated by IL-1Ra. On day 11, IL-12 remained down-regulated (PBS, 1.01±0.01 pg/mg vs. IL-1Ra, 0.63±.02 pg/mg) whereas IL-2 (PBS, 0.14±0 pg/mg vs. IL-1Ra, 0.10±0.04 pg/mg) and IFN- γ (PBS,.21 pg/mg vs. IL-1Ra,.19±.01 pg/mg) was not significantly different between treatments. Of the anti-inflammatory cytokines measured, IL-10 was significantly up-regulated by IL-1Ra treatment at day 5 (Fig. 5f; PBS, 0±0.00 pg/mg vs. IL-1Ra, 0.41±0.09 pg/mg), but not day 11 (PBS, 0.36±.00 pg/mg vs. 0.36±0.01 pg/mg). IL-6 (Fig. 5g) was not significantly different between treatments. Levels of GM-CSF, TNF-α, and IL-4 were below the detectable sensitivity range.

### Mechanical Testing

**Figure 6:**
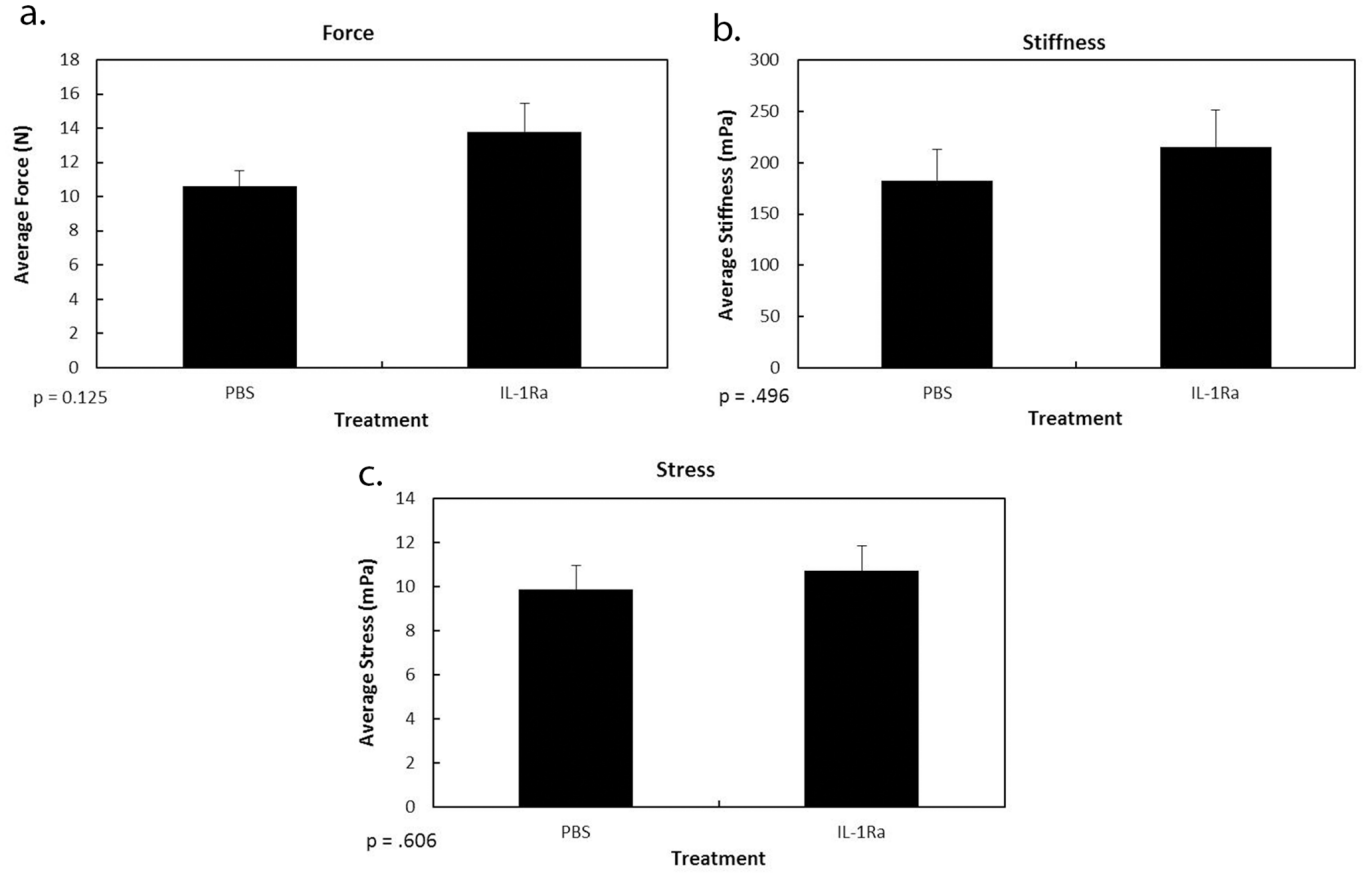
Mechanical results of the healing MCL after IL-1Ra treatment. Graphs show failure force (a), stiffness (b), and failure stress (c) of the MCL 11 days post-injury after PBS or IL-1Ra treatment. No significance was observed in any tested parameter (p>.05). Results are expressed as mean ± S.E.M.

To determine if IL-1Ra treatment affected ligament function, the day 11 MCLs (Fig. 6) were mechanically tested. Ligament failure force, failure stress, and stiffness were measured. No significance difference was found between IL-1Ra-treated specimens and the PBS control for any parameter tested (p>0.05). These results therefore suggest that IL-1Ra treatment did not reduce the mechanical properties of the healing ligament or inhibit functional recovery.

## Discussion

This study helps elucidate the role of cytokines in ligament healing. Interleukin expression after injury is a critical regulator of inflammation, healing and downstream scar formation. The present study identified the interleukins significantly modulated during MCL healing. Of the interleukins identified, components of the IL-1 family were significantly up-regulated. The impact of IL-1 was further demonstrated after exogenous administration of IL-1Ra to the injured MCL at the time of surgery to inhibit IL-1 activation. This resulted in decreased pro-inflammatory cytokines (IL-12, IL-2), myofibroblasts, and proliferating cells, as well as increased anti-inflammatory cytokines (IL-10), endothelial cells/blood vessel lumen, M2 macrophages, and granulation tissue size. These results support the concept that IL-1Ra modulates some MCL-localized granulation tissue components and cytokine production to create a transient environment that is less inflammatory. Of note, however, these changes in the inflammatory cascade did not significantly compromise or improve mechanical outcome, nor did they sustain a more regenerative healing response.

Microarray analysis of the normal healing MCL revealed significant changes in interleukin expression. Of interest, *Il1b* and *Il1ra* were significantly up-regulated after injury. IL-1 plays a central role in cell growth, tissue repair and chronic inflammatory diseases, and has emerged as a critical player in inflammation and tissue destruction of arthritis models. [14]–[17] Receptors for IL-1 are found on the macrophages, fibroblasts, T and B lymphocytes, enabling IL-1β to elicit both an inflammatory and fibrotic response. Indeed, previous studies have identified IL-1β during both M1-macrophage-predominated inflammation and myofibroblast/matrix deposition-controlled fibrogenesis [18] The naturally occurring inhibitor to IL-1 is IL-1Ra. IL-1Ra is able to bind to the IL-1R with similar affinity as IL-1α and IL-1β and inhibit IL-1 activity. Past reports have indicated that IL-1Ra suppresses pathologies related to the abnormal increase of IL-1 in rheumatoid arthritis, osteoarthritis, and intervertebral disc degeneration.[19]–[25] Although IL-1Ra binding affinity to IL-1R is high, a 10–100 molar excess of IL-1Ra is required to inhibit IL-1 activity. [26], [27] Taken together, the results suggest that excess administration of IL-1Ra in our protocol should inactivate IL-1 signaling which in turn, should modulate macrophage-stimulated inflammation and/or myofibroblast-induced fibrosis in our healing MCLs.

The increase in M2 macrophages after IL-1Ra administration suggests that IL-1Ra stimulated macrophage polarization to the M2 phenotype. A previous report demonstrated fibrotic and macrophage polarization effects using the IL-1 signal transducer, MyD88, knockout mouse model. [28] MyD88 normally induces an M1 polarization, but a lack of MyD88 altered macrophage polarization towards an anti-inflammatory M2 phenotype. [28] The current results likewise suggest that inhibition of IL-1 activation induces an M2 macrophage phenotype. The Multiplex data further suggest that IL-1Ra stimulate an anti-inflammatory response as indicated by the day 5 increase in anti-inflammatory IL-10 and concomitant decrease in pro-inflammatory IL-2 and IL-12.

IL-1 has been identified as a strong promoter of angiogenesis. [26], [29] However, IL-1inhibition in the current study actually increased endothelial cells and blood vessel lumen number. This increase may result from the IL-1Ra-induced increase in M2 macrophages. Indeed, IL-1 failed to stimulate an angiogenic response by endothelial cells without the presence of inflammatory cells, *in vitro.* [30] Macrophages play an important role in the angiogenic process, by facilitating vascular permeability for endothelial cell migration and promoting endothelial cell proliferation. Several studies have indicated that the M2 macrophages promote angiogenesis. The increase in M2 macrophages and decrease in proliferating cells in the current study suggests M2 macrophages promote endothelial cell migration and permeability rather than endothelial proliferation. Because endothelial cells were not increased from control values at day 11 suggests the M2 macrophages alone cannot sustain an endothelial cell response.

The day 5 decrease in myofibroblasts and subsequent increase in granulation tissue size suggests an influence of IL-1Ra on wound contraction. Previous studies have reported an increase in mitogenic activity and differentiation of myofibroblasts after IL-1 β treatment. [31]–[33] Blocking IL-1 activation via IL-1Ra may thereby inhibit fibroblast proliferation and/or differentiation to myofibroblasts. The early decrease in myofibroblasts and proliferating cells in the current study suggests IL-1Ra bound to the fibroblast-localized IL-1 receptors and inhibited fibroblast proliferation and possibly fibroblast differentiation into myofibroblasts. Myofibroblasts function in early wound contraction and later scar formation. The larger granulation tissue size but insignificant change in type III collagen (a marker for scar formation) suggest that IL-1Ra inhibits myofibroblast-induced wound contraction, rather than scar formation. Taken together, these results suggest that IL-1Ra influences granulation tissue formation by modulating the myofibroblasts and M2-stimulated endothelial cell migration.

In contrast to a previous study that ablated macrophages during ligament healing [34], IL-1Ra administration had no negative impact on the ligament mechanical properties. The trend (p = 0.125) for the IL-1Ra to increase in failure load in the current study is, nevertheless, intriguing. Treatments were not specifically optimized for concentration requirements and half-life of IL-1Ra in order to induce an improved mechanical outcome downstream. IL-1 is a potent cytokine and 100 fold or greater levels of IL-1Ra over IL-1 are necessary to functionally inhibit the biological effects of IL-1 on target cells. [35] Additionally, the half-life of IL-1Ra is only 6–8 hours. Without additional IL-1Ra treatments, the direct impact of IL-1Ra would rapidly diminish and may not sufficiently affect downstream healing events. This interpretation explains both early and late results in this study, *i.e.* enough IL-1Ra was administered to elicit a biological response attenuating some inflammatory events, but the dose and half-life were not sufficient to improve “regenerative” healing and mechanical properties at day 11.

Overall, IL-1Ra altered the granulation tissue components of the healing ligament by stimulating an M2 macrophage and endothelial cell response while inhibiting proliferation of myofibroblasts. IL-1Ra may then have therapeutic potential to favorably alter the healing cascade. However, the single dose of IL-1Ra used in this study was not sufficient to maintain the regenerative response at levels of statistical significance. Due to the transient influence of IL-1Ra on most of the healing components assayed, a more sustained delivery of IL-1Ra should be explored.
